# *Trichophyton mentagrophytes* Typ VII: Kohortenstudie zu Patientencharakteristika, klinischen Merkmalen, Krankheitsverlauf und Therapie

**DOI:** 10.1111/ddg.15837_g

**Published:** 2025-12-11

**Authors:** Konstanze Kämmerer, Julia Huynh, Cornelia Deutsch, Stefanie May, Sertac Uyar, Alexander Nast, Christoph Zeyen, Ricardo Niklas Werner

**Affiliations:** ^1^ Klinik für Dermatologie Venerologie und Allergologie Division of Evidence‐Based Medicine in Dermatology (dEBM) Charité – Universitätsmedizin Berlin Corporate member of Freie Universität Berlin and Humboldt‐Universität zu Berlin; ^2^ Klinik für Dermatologie Venerologie und Allergologie Charité – Universitätsmedizin Berlin Corporate member of Freie Universität Berlin and Humboldt‐Universität zu Berlin; ^3^ Klinik für Dermatologie Venerologie und Allergologie Mykologisches Labor Charité – Universitätsmedizin Berlin Corporate member of Freie Universität Berlin and Humboldt‐Universität zu Berlin

**Keywords:** Dermatophytose, Kerion Celsi, Majocchi‐Granulom, sexuell übertragbare Krankheiten, *Trichophyton mentagrophytes* Genotyp VII, Tinea, Terbinafin, dermatophytosis, kerion celsi, Majocchi granuloma, sexually transmitted diseases, *Trichophyton mentagrophytes* genotype VII, tinea, terbinafine

## Abstract

**Hintergrund und Ziele:**

Sexuell übertragene Infektionen mit *Trichophyton mentagrophytes* Typ VII (TMVII) wurden aus mehreren Ländern berichtet. Ziel unserer Studie ist, epidemiologische und klinische Charakteristika zu identifizieren, um Prävention, Diagnosestellung und Behandlung zu verbessern.

**Patienten und Methodik:**

Es wurden Patienten mit kulturell bestätigter TMVII‐Infektion eingeschlossen, die zwischen 2020 und 2024 in der Hautklinik der Charité –Universitätsmedizin Berlin diagnostiziert wurden. Patientencharakteristika, Klinik und Behandlung wurden ausgewertet.

**Ergebnisse:**

Insgesamt wurden 51 Patienten eingeschlossen (96,1% männlich, Median: 34 Jahre), wobei 94,6% der männlichen Patienten angaben, Sex mit Männern zu haben. Von 47 mit bekanntem HIV‐Status lebten 19,1% mit HIV; 71,1% der HIV‐negativen Patienten gaben an, PrEP zu nutzen. Die Dauer der Diagnosestellung betrug 42,5 Tage (Median; Q1–Q3: 29,25–73,5). Atypische Morphologien wie eitrige oder infiltrierte subkutane Läsionen traten bei 82,4% auf; 96,1% hatten genitale, perianale oder periorale Manifestationen. Die mediane Dauer der systemischen Behandlung betrug 83,5 Tage (Q1–Q3: 57–110,25), ohne signifikante Assoziation mit Morphologie (*p*  =  0,69) oder HIV‐/PrEP‐Status (*p*  =  0,88).

**Schlussfolgerungen:**

Infektionen mit TMVII treten häufig mit atypischer Morphologie auf und sollten in die Differenzialdiagnose genitaler, perianaler und perioraler Dermatosen einbezogen werden, insbesondere bei Männern mit wechselnden Sexpartnern. Oft sind lange Behandlungszeiten erforderlich, um eine Heilung zu erreichen und Rezidive zu vermeiden.

## EINLEITUNG

Dermatophyten verursachen Infektionen der Haut und Hautanhangsgebilde, die eine Reihe unterschiedlicher klinischer Erscheinungsformen annehmen können. Tinea in der Nähe von Schleimhäuten – etwa im Genital‐, Inguinal‐, Perianal‐, Gluteal‐ oder Perioralbereich – wurde mit verschiedenen Dermatophyten assoziiert.^1^, ^2^, ^3^, ^4^, ^5^, ^6^, ^7^, ^8^, ^9^, ^10^ Fälle von Autoinokulation aus anderen Körperregionen^4^, ^8^ oder anderweitigen Expositionen^6^, ^10^, ^11^ wurden berichtet, Infektionen in den genannten anatomischen Arealen legen jedoch eine potenzielle sexuelle Übertragung nahe.^2^, ^3^, ^5^, ^7^

*Trichophyton mentagrophytes* (TM), der als zoophiler Dermatophyt häufig mit entzündlichen und purulenten Läsionen einhergeht, wurde gehäuft bei Patienten mit Verdacht auf sexuelle Übertragung festgestellt.^12^, ^13^, ^14^ Im Jahr 2019 wurde TM‐Genotyp VII (TMVII) als eigenständige, bei Patienten mit entzündlicher anogenitaler Tinea gehäuft auftretende genetische Variante identifiziert.^15^

*Trichophyton mentagrophytes* Genotyp VII wird durch Sequenzierung der Internal‐Transcribed‐Spacer (ITS)‐Region von anderen TM‐Genotypen unterschieden. In der Pilzkultur lässt sich TMVII anhand charakteristischer phänotypischer Merkmale von anderen TM‐Genotypen abgrenzen – hierzu gehören sein schnelles Wachstum und die dunkle Pigmentierung auf der Rückseite der Kolonien, zusätzlich zur typischen Morphologie von TM.^16^, ^17^ Die mikroskopische Untersuchung kann weitere Hinweise liefern, wie etwa eine geringere Anzahl oder das Fehlen von Makrokonidien sowie ein häufigeres Vorkommen von Chlamydosporen im Vergleich zu anderen TM‐Genotypen. In unserem mykologischen Labor wurde der kulturbasierte phänotypische Ansatz zur Identifikation von TMVII mittels ITS‐Sequenzierung intern validiert und wird routinemäßig in der Diagnostik angewandt.

Seit seiner genetischen Charakterisierung wurde TMVII zunehmend bei Patienten mit genitaler, perianaler und perioraler Tinea festgestellt.^17^ Wissenschaftliche Berichte deuten auf eine endemische und anthropogene Übertragung in mehreren Regionen hin: 37 Fälle von entzündlichen pubogenitalen TMVII‐Infektionen wurden zwischen 2016 und 2017 bei männlichen und weiblichen Patienten in Berlin identifiziert, wobei die meisten von ihnen keine Anamnese von Auslandsreisen oder Tierkontakten aufwiesen.^15^ In Paris wurden 13 Fälle mit Läsionen in der Bart‐, Genital‐ und Glutealregion beschrieben, hauptsächlich bei Männern, die Sex mit Männern haben (MSM), was auf eine Übertragung innerhalb sexueller Netzwerke hindeutet.^3^ Weitere Fälle von TMVII‐Infektionen unter MSM wurden aus New York,^18^, ^19^ Paris^20^ und Barcelona^21^ berichtet.

Klinisch stellt sich die Tinea corporis typischerweise in Form oberflächlicher erythematöser, schuppender Plaques mit Randbetonung dar (Ringelflechte).^22^ Infektionen mit dem TMVII‐Genotyp zeigen jedoch häufig eine vom typischen Erscheinungsbild abweichende, stark inflammatorische und purulente Morphologie.^3^, ^15^, ^17^, ^23^ Neben der atypischen klinischen Manifestationen kann auch die ungewöhnliche anatomische Lokalisation zu einer erschwerten und verzögerten Diagnose dieser Dermatophytose beitragen.^3^ Zudem erfordert die Behandlung häufig eine verlängerte Anwendung systemischer Antimykotika, und ein vorzeitiges Therapieende kann mit Rezidiven einhergehen.

Zu den epidemiologischen und klinischen Aspekten sowie den verfügbaren therapeutischen Optionen liegen bislang nur wenige Daten vor. Ziel der vorliegenden Studie ist es, Erkenntnisse zu den epidemiologischen und klinischen Merkmalen, dem Krankheitsverlauf sowie den Behandlungsergebnissen von TMVII‐Infektionen zu gewinnen.

## PATIENTEN UND METHODIK

### Studienaufbau, ‐setting und ethische Aspekte

In dieser nichtinterventionellen Kohortenstudie wurden Patienten eingeschlossen, die aufgrund einer kulturell bestätigten TMVII‐Infektion zwischen Januar 2020 und August 2024 an der Klinik für Dermatologie, Venerologie und Allergologie der Charité – Universitätsmedizin Berlin behandelt wurden. Patienten, die vor Juli 2023 behandelt wurden, wurden retrospektiv eingeschlossen. Die Datenerhebung erfolgte anhand der medizinischen Dokumentation in den Patientenakten. Ab Juli 2023 erfolgte ein prospektiver Einschluss, und Patienten nahmen nach Erteilung der schriftlichen informierten Einwilligung zusätzlich an strukturierten Interviews teil. Vor Beginn der Datenerhebung wurde eine Genehmigung durch die institutionelle Ethikkommission eingeholt (EA4/209/23).

### Probanden

Patienten wurden eingeschlossen, wenn sie eine kulturell‐morphologisch bestätigte TMVII‐Infektion hatten. Für die Pilzkultur wurden Hautschuppen, enthaarte Haarschäfte oder Pus aus Läsionen auf modifizierte Dermatophyten‐Agarplatten (Sifin, Berlin, Deutschland; Zusammensetzung pro Liter: Glucose 20,0 g, Chloramphenicol 0,1 g, Cycloheximid 0,4 g, Pepton 10 g, Agar 16 g) inokuliert und für mindestens vier Wochen inkubiert. Die TMVII‐Diagnose basierte auf der makroskopischen und mikroskopischen phänotypischen Beurteilung, wie in der Einleitung beschrieben. Nur Patienten im Alter von ≥ 18 Jahren waren teilnahmeberechtigt.

### Variablen

**ABBILDUNG 1:**
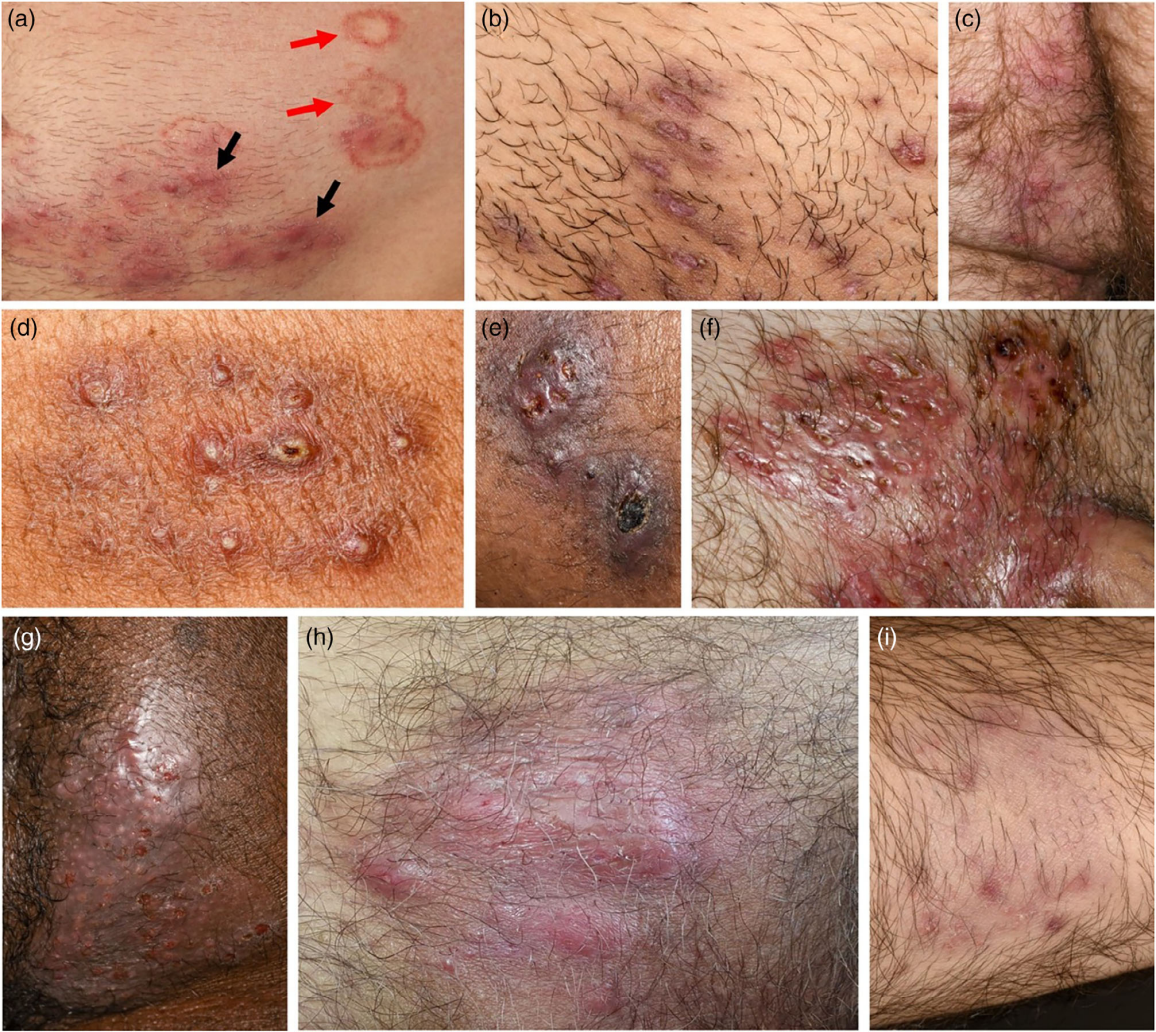
Klinische Präsentationen von Infektionen mit *Trichophyton mentagrophytes* Typ VII. (a) Typische Läsionen einer Tinea corporis (oberflächliche, scharf begrenzte erythematöse Plaques; rote Pfeile) und atypische subkutane follikuläre Papeln und Pusteln (schwarze Pfeile) am Unterbauch und Mons pubis. (b–d) Subkutane, teils follikelassoziierte Papeln und Pusteln im Genital‐, Perianal‐ und Glutealbereich bei drei verschiedenen Patienten. (e–h) Zu Knoten und Plaques konfluierende subkutane Papeln und Pusteln. (i) Umschriebene Alopezie am Unterarm.

Es wurden Daten zu den Patientencharakteristika, klinischen Merkmalen, Krankheitsverlauf, der Behandlung und dem Folgezustand gesammelt. Zu den Patientencharakteristika gehörten Alter, Geschlecht, sexuelle Orientierung, Partnerschaftsstatus, Anzahl der Sexpartner, Tierkontakt und der vermutete Infektionsmodus. Zusätzlich wurden der HIV‐Status, Nutzung von HIV‐Präexpositionsprophylaxe (PrEP), Immunsuppression und die Anamnese sexuell übertragbarer Infektionen (STI) erfasst. Klinische Merkmale umfassten Schmerz und Juckreiz, jeweils bewertet auf einer numerischen Ratingskala (NRS) von 0 bis 10, die anatomische Lokalisation, die Läsionsmorphologie (Tabelle 1, Abbildung 1) und regionale Lymphadenopathie. Daten zum Krankheitsverlauf umfassten die Zeit vom Symptombeginn bis zur Diagnosestellung oder zum Beginn der systemischen antimykotischen Behandlung (je nachdem, was früher eintrat), die Gesamtdauer der systemischen Behandlung (gegebenenfalls einschließlich Medikamentenwechsel, jedoch ohne Therapiepausen) sowie die Dauer der initialen Behandlung bei Patienten, bei denen die systemische Therapie nach Unterbrechung erneut begonnen wurde. Behandlungsdaten umfassten die Medikation, die Reihenfolge und Kombination der systemischen und topischen antimykotischen Therapien. Beim letzten Follow‐up wurden Folgezustände wie Narbenbildung, Hyperpigmentierung, Schmerz und Juckreiz erfasst.

**TABELLE 1:** Klassifikation der Läsionsmorphologie.

Morphologie	Beschreibung	Klassifikation
Oberflächlich	Oberflächliche, scharf begrenzte erythematöse Plaques mit Randbetonung und Schuppung (Beispiel, siehe Abbildung 1a, rote Pfeile)	Typische Morphologie einer Tinea corporis
Purulent	Purulente Läsionen, z. B., follikuläre Pusteln (Abbildung 1d) oder purulente Plaques (Abbildung 1g)	Atypische Morphologie
Subkutan	Subkutane, tief infiltrierende Papeln (Abbildung 1a, schwarze Pfeile), Knoten (Abbildung 1h) oder Plaques (Abbildung 1f, g)	Atypische Morphologie

### Datenquellen und Messmethoden

Einzuschließende Patienten wurden durch eine systematische Durchsicht der Patientenakten (retrospektiver Einschluss bis Juni 2023) und durch die behandelnden Ärzte der Klinik (prospektiver Einschluss ab Juli 2023) identifiziert. Die Daten wurden den Patientenakten entnommen. Mit den prospektiv aufgenommenen Patienten wurden zusätzlich strukturierte Interviews durchgeführt, um für die oben genannten Variablen umfassende und standardisierte Daten zu erheben. Zur Gewährleistung der Vertraulichkeit wurden allen Teilnehmern Pseudonyme zugewiesen, die in einer verschlüsselten, passwortgeschützten Datei gespeichert und getrennt von den Studiendaten aufbewahrt wurden.

### Datenanalyse

Kategorische Variablen wurden als Häufigkeiten und Prozentsätze zusammengefasst. Kontinuierliche Variablen wurden, je nach Verteilung der Daten, unter Verwendung von Mittelwerten und Standardabweichungen oder Medianen und Quartilen (Q1, Q3) beschrieben. Die Verteilung der numerischen Daten wurde mit Hilfe von Boxplots, Histogrammen und dem Kolmogorov‐Smirnov‐Test bewertet. Der Fisher's‐exact‐Test wurde verwendet, um Zusammenhänge zwischen kategorialen Variablen zu untersuchen. Zur Quantifizierung der Assoziationsstärke zwischen anatomischer Lokalisation und Läsionsmorphologie einerseits sowie subjektiven Symptomen und Folgezuständen andererseits wurden das relative Risiko (RR) und die entsprechenden 95%‐Konfidenzintervalle (95%‐KI) berechnet. Der Mann‐Whitney‐U‐Test und der Kruskal‐Wallis‐Test wurden verwendet, um Unterschiede in der Diagnose‐ und Behandlungsdauer basierend auf Morphologie und HIV/PrEP‐Status zu bewerten. Statistische Analysen wurden mit R und RStudio (Version 2024.04.2 + 764) durchgeführt. Der Signifikanzschwellenwert wurde auf α  =  0,05 festgelegt. Zur Kontrolle der *false discovery rate* wurde das Benjamini‐Hochberg‐Verfahren (BH) angewandt. Sowohl unadjustierte als auch BH‐adjustierte p‐Werte werden berichtet.^24^, ^25^

## ERGEBNISSE

### Demografische Merkmale

Es wurden 51 Patienten eingeschlossen, davon 34 retrospektiv und 17 prospektiv. Das Alter der Patienten reichte von 24 bis 69 Jahren (Mittelwert: 37,2 Jahre, Standardabweichung [SD]: 11,2; Median: 34 Jahre, Q1–Q3: 29,5–40 Jahre). Neunundvierzig Patienten (96,1%) waren männlich. Informationen zur sexuellen Orientierung lagen für 37 männliche Patienten vor, von denen 35 (94,6%) Sex mit Männern berichteten, während zwei (5,4%) angaben, ausschließlich Sex mit Frauen zu haben. Beide Frauen gaben an, Sex mit Männern zu haben. Die demografischen Merkmale sind in Tabelle 2 dargestellt.

**TABELLE 2:** Demografische Patientencharakteristika.

**Alter (Jahre), n = 51**		
Median (Q1–Q3)	34	(29.5–40)
Spannweite	24–69	
**Geschlecht, n = 51**		
	*n*	*%*
Cis‐Mann	49	96.1
Cis‐Frau	2	3.9
**Sexuelle Orientierung, n = 39**		
	*n*	*%*
MSW	2	5.1
MSM	34	87.2
WSM	2	5.1
MSMW	1	2.6
**Anzahl Sexpartner (6 Monate vor Beginn der Symptome), n = 22**		
Median (Q1–Q3)	7	(1.0–21.25)
Spannweite	1–100	

*Abk*.: Q1, erstes Quartil; Q3, drittes Quartil; MSW, Männer, die Sex mit Frauen haben; MSM, Männer, die Sex mit Männern haben; WSM, Frauen, die Sex mit Männern haben; MSMW, Männer, die Sex mit Männern und Frauen haben

Unter den 47 Patienten mit Informationen zum HIV‐Status lebten neun (19,1%) mit HIV (people living with HIV; PLWH). Einer dieser Patienten wurde im Zusammenhang mit der vorliegenden Pilzinfektion neu diagnostiziert. Dieser Patient wies eine nachweisbare Viruslast und eine CD4‐Zahl von 280/µl auf, während alle anderen PLWH eine nicht nachweisbare Viruslast hatten. Unter den 38 HIV‐negativen Patienten gaben 27 (71,1%) an, PrEP zu verwenden. Abgesehen von HIV lag keine bekannte Immunsuppression vor.

Die Anzahl der Sexpartner in den sechs Monaten vor Beginn der Symptome variierte zwischen 1 und 100 (Median: 7, Q1–Q3: 1–21,25, n  =  22). Unter den 31 Patienten, die Angaben zur Anamnese sexuell übertragbarer Infektionen machten, erklärten 29 (93,5%), jemals eine STI gehabt zu haben. Eine Lebenszeit‐Diagnose von Chlamydien, Gonorrhoe, Syphilis und Hepatitis C wurde von 65,4%, 59,3%, 56,7% und 3,8% der Patienten angegeben. Es wurde eine mediane Zahl von einer STI‐Diagnose in den 12 Monaten vor der Diagnose der Pilzinfektion berichtet (Spannweite 0–6, Q1–Q3: 1–2, n  =  26). Es bestand eine Korrelation zwischen der Anzahl der Sexpartner und der STI‐Diagnosen, die jedoch nach BH‐Adjustierung nicht signifikant war (ρ  =  0,601, *p*  =  0,008, BH‐adjustiertes *p*  =  0,088, n  =  18).

Vier Teilnehmer berichteten, einen Sexpartner mit vergleichbaren Läsionen oder einer bekannten Pilzinfektion gehabt zu haben. Von 28 Patienten berichteten acht (28,6%) über Kontakt mit Hunden oder Katzen, wobei nur einer von ihnen erythematöse Hautläsionen an seinem Hund beobachtet hatte.

### Klinische Präsentation der akuten Infektion

Vierundzwanzig Patienten (47,1%) wiesen eine einzige betroffene anatomische Region auf. Fünfzehn (29,4%), acht (15,7%) und vier (7,8%) Patienten hatten zwei, drei beziehungsweise vier betroffene Regionen. Bemerkenswerterweise hatten 49 Patienten (96,1%) ihre Infektionen an einer perianalen, genitalen und/oder fazialen Regionen lokalisiert. Weitere Details zur anatomischen Verteilung sind in Tabelle 3 aufgeführt.

**TABELLE 3:** Klinische Präsentation der Läsionen.

**Lokalisation, n = 51**	**n**	**%**
Genitale, perianale und/oder Gesichtsbeteiligung	49	96.1
Genital, pubisch	27	52.9
Perianal, gluteal	18	35.3
Perioral, Gesicht	25	49.0
Kopfhaut	4	7.8
Extremitäten/Rumpf	2	3.9

**Morphologie, n = 51**	**n**	**%**
Oberflächliche Läsionen	40	78.4
Ausschließlich oberflächliche Läsionen	9	17.6
Atypische Morphologie	42	82.4
Purulente Läsionen	40	78.4
Subkutane Läsionen	14	27.5
Ausschließlich atypische Läsionen	11	21.6

**Lymphadenopathie, n = 28**	**n**	**%**
Vorhanden	9	32.1
Nicht vorhanden	19	67.9

Unter den 51 Patienten wiesen lediglich neun (17,6%) ausschließlich oberflächliche Läsionen auf. Zweiundvierzig Patienten (82,4%) wiesen atypische Erscheinungsformen der Tinea auf, wie beispielsweise purulente Läsionen (40 Fälle, 78,4%) und/oder subkutane Knoten, Plaques oder Infiltrate (14 Fälle, 27,5%). Elf Patienten (21,6%) zeigten eine ausschließlich atypische Präsentation. Weitere Details zu den klinischen Präsentationen und Beispiele sind in Tabelle 3 und Abbildung 1 dargestellt.

Purulente Läsionen traten häufiger bei PrEP‐Nutzern (24 von 27; 88,9%) und PLWH auf (8 von 9; 88,9%), verglichen mit den anderen Teilnehmern (5 von 11; 45,5%). Diese Assoziation war jedoch nach BH‐Adjustierung nicht signifikant (Fisher's‐exact‐Test: p  =  0,013, BH‐adjustiertes p  =  0,072). Ansonsten unterschied sich die Morphologie nicht signifikant in Abhängigkeit vom HIV‐Status oder PrEP‐Nutzung.

Subjektive Symptome sind in Tabelle 4 dargestellt. Schmerz wurde von 31 der 39 Patienten (79,5%) berichtet. Der mediane NRS‐Wert betrug 7 (Q1–Q3: 4–8, Spannweite 1–8, n  =  17). Dreißig von 36 (83,3%) gaben Juckreiz an, mit einem medianen NRS‐Wert von sieben (Q1–Q3: 4–8, Spannweite 2–10, n  =  18). Obwohl Teilnehmer mit atypischer Morphologie ein höheres Risiko für das Auftreten von Schmerzen (87,1%) hatten als diejenigen mit ausschließlich oberflächlichen Läsionen (50,0%), wurde keine statistisch signifikante Assoziation gefunden (RR  =  1,74, 95%‐KI: 0,86–3,53). Ebenso war der Anteil der Patienten, die Schmerzen berichteten, bei anogenitaler Lokalisation höher als bei anderen Lokalisationen, jedoch bestand keine signifikante Assoziation (RR  =  1,25, 95%‐KI: 0,77–2,04). Es wurden keine Unterschiede in der Häufigkeit von Schmerzen oder Juckreiz in Abhängigkeit vom HIV‐Status oder PrEP‐Nutzung festgestellt (Schmerz: p  =  0,86, BH‐adjustiertes p > 0,99, Juckreiz: p > 0,99, BH‐adjustiertes p > 0,99, Fisher's‐exact‐Test).

**TABELLE 4:** Subjektive Symptome.

**Schmerz, n = 39**	**n**	**%**
Schmerz vorhanden	31	79.5
**Schmerz, numerische Bewertungsskala (0–10), n = 17**		
Median (Q1–Q3)	7	(4–8)
Spannweite	1–8	

**Juckreiz, n = 36**	n	%
Juckreiz vorhanden	30	83.3
**Juckreiz, numerische Bewertungsskala (0–10), n = 18**		
Median (Q1–Q3)	7	(4–8)
Spannweite	2–10	

*Abk*.: Q1, erstes Quartil; Q3, drittes Quartil

Die mediane Zeit vom Auftreten der ersten Symptome bis zur Diagnosestellung oder zum Beginn der antimykotischen Behandlung betrug 42,5 Tage (Q1–Q3: 29,25–73,5, Spannweite 12–294 Tage, n  =  48). Dies unterschied sich nicht signifikant zwischen Patienten mit purulenten oder subkutanen Läsionen und denen mit ausschließlich oberflächlichen Läsionen (*p*  =  0,15, BH‐adjustiertes *p*  =  0,34, Mann‐Whitney‐U‐Test) sowie zwischen PrEP‐Nutzern, PLWH und anderen Teilnehmern (*p*  =  0,44, BH‐adjustiertes *p*  =  0,80, Kruskal‐Wallis‐Test). Die Dauer bis zur Diagnose korrelierte moderat positiv mit dem Alter (ρ  =  0,35, *p*  =  0,014, BH‐adjustiertes *p*  =  0,051, n  =  48) und moderat negativ mit der Anzahl betroffener anatomischer Lokalisationen (ρ  =  ‐0,35, *p*  =  0,015, BH‐adjustiertes *p*  =  0,041, n  =  48).

### Behandlung und Krankheitsverlauf

Bis auf einen erhielten alle Patienten (98%) eine topische Behandlung, entweder zusätzlich zur systemischen Therapie oder als alleinige Behandlung. Sechsundzwanzig Patienten (52,0%) erhielten eine topische Substanz, während 24 (48,0%) mehr als eine topische Substanz erhielten, entweder in Kombination oder nacheinander. Einundvierzig Patienten (82,0%) wurde Ciclopiroxolamin verschrieben, zwölf (24,0%) Ketoconazol, elf (22,0%) Miconazol, zehn (20,0%) Clotrimazol, zwei (4,0%) Econazol und einem (2,0%) topisches Terbinafin.

Bei 46 der 49 Patienten (93,9%) wurde eine systemische Behandlung eingeleitet. Während 37 der 46 systemisch behandelten Patienten (80,4%) nur ein systemisches Medikament erhielten, wurden bei neun Patienten (19,6%) zwei oder mehr Substanzen nacheinander angewandt. Zur Initiierung der systemischen Therapie wurden Terbinafin, Itraconazol und Fluconazol bei 38 (77,6%), sieben (14,3%) beziehungsweise einem (2,0%) Patienten eingesetzt. Vier von 38 (13,2%) Patienten, die mit Terbinafin behandelt wurden, wurden aufgrund vermuteter behandlungsbedingter unerwünschter Wirkungen (2 Fälle) oder unzureichendem Behandlungserfolg (2 Fälle) auf Itraconazol umgestellt. Ein Patient wurde nach einer kurzen Behandlung mit Terbinafin aufgrund unzureichenden Ansprechens von einem externen Arzt auf Fluconazol umgestellt (Dauer unklar). Dieser Patient sprach nach einer erneuten Behandlung mit Terbinafin adäquat an. Drei der sieben Patienten, die mit Itraconazol behandelt wurden (42,9%), wurden aufgrund unzureichender Wirkung auf Terbinafin umgestellt. Der einzige Patient, der mit Fluconazol behandelt wurde, wurde nach einer 42‐tägigen Behandlungsdauer aufgrund unzureichenden Ansprechens auf Itraconazol umgestellt. Unerwünschte Wirkungen (UW) wurden bei acht Patienten (17,4%) dokumentiert (Tabelle 5).

**TABELLE 5:** Unerwünschte Wirkungen (UW).

**Gesamtzahl UW**	**8**	**Unter der Behandlung mit**
Erhöhung der Leberenzyme	1	Terbinafin
Gastrointestinal	3	Terbinafin
Hypogeusie	1	Terbinafin
Tagesmüdigkeit	1	Terbinafin
Arzneimittelexanthem^*^	1	Terbinafin
Phototoxische Reaktion^*^	1	Terbinafin

* Absetzen / Umstellung der Therapie

Die mediane Gesamtdauer der systemischen Therapie (ohne therapiefreie Intervalle) betrug 83,5 Tage (Q1–Q3: 57,0–110,25; Bereich: 28–226 Tage; n  =  40). Es zeigten sich keine signifikanten Unterschiede in der Behandlungsdauer zwischen Patienten mit ausschließlich oberflächlichen Läsionen und jenen mit purulenten oder subkutanen Manifestationen (*p*  =  0,69; BH‐adjustiertes *p*  =  0,95; Mann‐Whitney‐U‐Test) sowie in Abhängigkeit von HIV‐Status und PrEP‐Nutzung (*p*  =  0,88; BH‐adjustiertes *p*  =  0,97; Kruskal‐Wallis‐Test). In neun Fällen wurde die systemische Therapie unterbrochen; bei sechs dieser Patienten erfolgte die Unterbrechung nach initialer klinischer Abheilung mit anschließendem Rezidiv. In dieser Subgruppe lag die mediane Dauer der ersten Behandlungsphase bei 42 Tagen (Q1–Q3: 34,5–95,5; Bereich: 27–122 Tage). Insgesamt betrug der mediane Zeitraum zwischen Symptombeginn und letztem Follow‐up 152 Tage (Q1–Q3: 93,5–295,5; n  =  37).

### Folgezustände beim Follow‐up

Die mediane Zeitspanne zwischen dem Ende der systemischen Therapie und der letzten Nachuntersuchung betrug 4,5 Tage (Q1–Q3: –3,5–45; n  =  34). Für 34 Patienten lagen Daten zum klinischen Hautbefund und Folgezuständen am Therapieende beziehungsweise beim Follow‐up vor. Bei 25 (73,5 %) bestanden weiterhin klinisch sichtbare Hautveränderungen. Am häufigsten wurden postinflammatorische Hyperpigmentierungen beobachtet (13 Fälle, 38,2 %), gefolgt von Erythemen (9 Fälle, 26,5 %) sowie Narben oder Alopezie (6 Fälle, 17,7 %). Pruritus wurde von einer von 24 Personen (4,2 %) angegeben; Schmerzen wurden nicht berichtet.

Im Vergleich zu den Patienten mit ausschließlich oberflächlichen Läsionen war das Risiko für persistierende Hautbefunde bei denjenigen mit purulenten oder subkutanen Läsionen leicht erhöht, jedoch ohne statistische Signifikanz (RR: 1,36, 95%‐KI: 0,69–2,67). Es wurden keine klinisch oder statistisch signifikanten Unterschiede in Bezug auf das Auftreten von persistierende Hautbefunden in Abhängigkeit vom HIV‐Status oder PrEP‐Nutzung festgestellt (p  =  0,49, BH‐adjustiertes p  =  0,77, Fisher's‐exact‐Test).

## DISKUSSION

Diese nichtinterventionelle Kohortenstudie präsentiert epidemiologische und klinische Daten von Patienten mit kulturell bestätigter TMVII‐Infektion, die an einer Universitätshautklinik in Berlin behandelt wurden. Ziel war es, durch die Erhebung und Darstellung von Patientencharakteristika, klinischer Präsentation, Krankheitsverlauf und Behandlungsergebnissen die Forschungslage zu diesem relativ neuen Dermatophyten zu verbessern. Die Ergebnisse stützen die zunehmenden Hinweise auf eine endemische Transmission, bei der die sexuelle Übertragung eine dominante Rolle spielt. Von Relevanz sind die häufig atypischen klinischen Präsentationen mit purulenten und subkutanen Läsionen sowie die langen Behandlungsdauern, die erforderlich sind, um die mykologische Heilung zu erreichen.

Wie in kürzlich veröffentlichten Fallserien beschrieben,^3^, ^18^, ^19^ wurde TMVII auch in unserer Kohorte überwiegend von sexuell aktiven MSM erworben. Neben der anatomischen Lokalisation der Befunde, wobei mehr als 95% der Teilnehmer Läsionen in der Nähe von Schleimhäuten aufwiesen, stützen mehrere epidemiologische Merkmale zusätzlich die Annahme einer sexuellen Übertragung. Dazu gehören etwa die hohe Zahl an Sexpartnern, anamnestisch multiple STI sowie der Anteil an PrEP‐Nutzern und Personen mit bekannter HIV‐Infektion. Obwohl Infektionen mit TMVII im Vergleich zu anderen STI weiterhin sehr selten sind, weist das epidemiologische Muster dieser Infektionen bei MSM Ähnlichkeiten mit den Beobachtungen bei anderen STI wie Syphilis oder Mpox auf.

Zusätzlich zu verhaltens‐ und netzwerkbezogenen Faktoren, wie der Einbindung in sexuelle Netzwerke mit hoher epidemiologischer Belastung durch sexuell übertragbare Infektionen, könnte der hohe Anteil von MSM mit mehreren Partnern und häufiger STI‐Anamnese auch auf eine erhöhte biologische Suszeptibilität hinweisen. Potenziell begünstigende Faktoren umfassen mikroskopische Hautläsionen (Mikrotraumata) sowie Veränderungen des Hautmikrobioms infolge wiederholter Antibiotikaanwendungen. Die Wirt‐Erreger‐Interaktionen im Kontext sexuell übertragbarer Pilzinfektionen sind bislang nur unzureichend erforscht. Der beobachtete Zusammenhang mit HIV‐Infektion und PrEP‐Nutzung könnte teilweise auch durch eine erhöhte Inanspruchnahme medizinischer Versorgungsangebote erklärt werden, was zu einer früheren Diagnosestellung von TMVII‐Infektionen führen könnte. Angesichts des hohen Anteils ausgeprägter symptomatischer Verläufe ist jedoch davon auszugehen, dass ein Großteil der Betroffenen auch unabhängig von ihrem Hintergrund ärztliche Hilfe in Anspruch genommen hätte.

Übereinstimmend mit früheren Berichten über Infektionen mit TMVII,^3^, ^15^, ^17^, ^23^ bestätigt unsere Kohorte, dass ein erheblicher Anteil der Patienten klinisch ungewöhnliche Manifestationen der Tinea corporis zeigte, etwa purulente, teils follikuläre Läsionen, sowie subkutane Plaques und Knoten – im Gegensatz zur typischen Morphologie mit Ausbildung randbetonter erythematöser und schuppender Plaques. Dies könnte eine Folge der engen genetischen Verwandtschaft von TMVII mit anderen TM‐Genotypen zoophilen Ursprungs sein.^15^ Eine stark entzündliche Reaktion ist bei Pilzinfektionen charakteristisch für zoophile Dermatophyten, wenn sie den Menschen infizieren.^1^ Das untypische klinische Bild kann zu einer Verzögerung der Diagnosestellung und Behandlungseinleitung führen. Während die mediane Zeit vom Auftreten der Symptome bis zur Diagnosestellung etwas weniger als anderthalb Monate betrug, dauerte die Diagnosestellung bei 25% der Patienten mehr als 73 Tage, in einem Fall sogar 290 Tage.

Unsere mediane Nachbeobachtungszeit von nahezu 5 Monaten zeigt, dass die Infektion viele Patienten über einen längeren Zeitraum hinweg betraf, was die Herausforderungen bei der Behandlung unterstreicht. Bei oberflächlicher Tinea corporis, die durch andere Dermatophyten verursacht wird, dauert die systemische Behandlung in der Regel 2 bis 4 Wochen. Die mediane Behandlungsdauer in unserer Kohorte betrug 12 Wochen, wobei ein Viertel der Patienten mehr als 15 Wochen benötigte. Dies entspricht den Ergebnissen einer Fallserie aus Paris, in der die systemische Therapie ebenfalls bis zu vier Monate lang verabreicht wurde.^3^ Bei einer kleinen Anzahl von Patienten, die nur 42 Tage (Median) behandelt wurden, musste die Behandlung aufgrund eines Rezidivs erneut begonnen werden.

Wir fanden keine signifikanten Unterschiede in der Behandlungsdauer zwischen atypischem und typischem Erscheinungsbild. Aufgrund der geringen Zahl von Patienten mit ausschließlich oberflächlichen Läsionen war die Aussagekraft dieses Vergleichs vermutlich eingeschränkt. Der hohe Anteil an Patienten mit subkutanen und purulenten Läsionen ist eine plausible Erklärung für die in unserer Kohorte beobachtete lange Behandlungsdauer. Eine zunehmende Terbinafin‐Resistenz bei Dermatophyten könnte ebenfalls zur beobachteten Entwicklung beitragen. Obwohl eine weitverbreitete Terbinafin‐Resistenz derzeit am häufigsten bei *Trichophyton indotineae* (ehemals: TM Genotyp VIII) beobachtet wird,^26^, ^27^, ^28^, ^29^ wurden auch Fälle mit reduzierter Terbinafin‐Suszeptibilität bei anderen Trichophyton‐Spezies berichtet. In unserer Kohorte wurden von 38 Patienten, die initial Terbinafin erhielten, nur zwei aufgrund eines vermuteten Behandlungsversagens auf Itraconazol umgestellt. Dies lässt darauf schließen, dass Terbinafin‐Resistenz in unserer Population keine erhebliche Rolle spielte. Dennoch sollte eine verringerte Empfindlichkeit gegenüber Antimykotika und das mögliche Aufkommen von Resistenzen bei TMVII nicht unbeachtet bleiben und bedarf weiterer Forschung. Eine kontinuierliche molekulare Überwachung von resistenzassoziierten Mutationen könnte wichtig sein, um Veränderungen der Suszeptibilität frühzeitig zu erkennen.^30^

Es zeigten sich keine signifikanten Unterschiede in der Behandlungsdauer oder ‐wirksamkeit zwischen PLWH und PrEP‐Nutzern. In diesem Zusammenhang ist hervorzuheben, dass bis auf eine Person alle PLWH in unserer Kohorte eine suffizient behandelte und gut kontrollierte HIV‐Infektion mit Viruslast unterhalb der Nachweisgrenze aufwiesen. Dies erklärt die vergleichbaren Behandlungsergebnisse. Wir konnten keine sonstigen plausiblen Faktoren identifizieren, wenngleich die Analyse potenzieller Störfaktoren aufgrund der relativ geringen Anzahl an PLWH eingeschränkt war.

Eine Limitation unserer Studie besteht darin, dass die Diagnose von TMVII‐Infektionen, wie in der Einleitung dargelegt, auf der phänotypischen Beurteilung der Pilzkulturen basierte. Wenngleich die ITS‐Sequenzierung den Goldstandard für die Speziesidentifikation und Genotypisierung darstellt,^17^, ^31^ wurden die spezifischen morphologischen Merkmale von TMVII auf Cycloheximid‐haltigem Dermatophyten‐Agar oder gewöhnlichem Sabouraud‐Dextrose‐Agar bereits in früheren Publikationen dokumentiert.^16^, ^17^ In unserem mykologischen Labor wird der kulturbasierte phänotypische Ansatz im Rahmen der Routinediagnostik konsequent angewandt und wurde in 31 vorherigen Isolaten intern validiert. In all diesen Fällen wurden Isolate, welche die für TMVII charakteristischen makro‐ und mikroskopischen Merkmale aufwiesen, durch ITS‐Sequenzierung bestätigt, ohne diskordante Ergebnisse. Dies unterstützt die Anwendung unseres kulturbasierten phänotypischen Ansatzes, allerdings wurde die diagnostische Genauigkeit bisher noch nicht im Rahmen einer Studie systematisch untersucht. Daher kann die Möglichkeit diagnostischer Fehlklassifikationen nicht vollständig ausgeschlossen werden. Hervorzuheben ist die ausgesprochene Konsistenz der epidemiologischen und klinischen Merkmale der eingeschlossenen Patienten und deren enge Übereinstimmung mit den Daten internationaler Studien, in denen TMVII durch ITS‐Sequenzierung bestätigt wurde.^18^, ^19^, ^20^, ^21^ Dies unterstützt zusätzlich die Validität unseres kulturbasierten diagnostischen Ansatzes. Wenngleich weitere Forschung zur Bestätigung der diagnostischen Genauigkeit erforderlich ist, könnte die kulturbasierte phänotypische Identifikation eine kostengünstige Alternative darstellen, um TMVII von anderen TM‐Genotypen zu unterscheiden.

Da die Teilnehmer in einem Universitätskrankenhaus rekrutiert wurden, ist es wichtig zu berücksichtigen, dass die Repräsentativität unserer Ergebnisse aufgrund einer möglichen Selektion schwerer oder refraktärer Fälle begrenzt sein könnte. Obwohl wir potenzielle Risikofaktoren identifiziert haben, lässt die kleine Stichprobengröße möglicherweise keine weitreichenden Schlussfolgerungen zu. Zukünftige Studien sollten Unterschiede zwischen atypischen und typischen Läsionen in Bezug auf Patientencharakteristika und Behandlungsergebnisse untersuchen.

Es ist wichtig, TMVII in die Differenzialdiagnose bei genitalen, perianalen und perioralen Hautläsionen aufzunehmen, besonders bei Risikogruppen wie MSM mit mehreren Partnern. Es ist notwendig, das Bewusstsein für diesen aufkommenden Erreger bei Klinikern und betroffenen Communities zu fördern, um eine frühzeitige Diagnose, fundierte Beratung und effektive Behandlung zu ermöglichen. Ein besseres Verständnis seiner epidemiologischen und klinischen Merkmale kann ein angemessenes klinisches Management unterstützen und zur Infektionskontrolle beitragen.

## DANKSAGUNG

ChatGPT (OpenAI, Version GPT‐4o mini) wurde zur Unterstützung bei der Übersetzung und sprachlichen Überarbeitung des Manuskripts verwendet.

Open access Veröffentlichung ermöglicht und organisiert durch Projekt DEAL.

## INTERESSENKONFLIKT

Keiner.
